# Medical Society of the State of New York

**Published:** 1898-03

**Authors:** 


A Monthly Review of Medicine and Surgery.

*EDITORS:*

THOMAS LOTHROP, M. D. - - WM. WARREN POTTER, M. D.

All communications, whether of a literary or business nature, should be addressed to the managing editor: 284 FRANKLIN STREET, BUFFALO, N. Y.

VOL. XXXVII. MARCH, 1898. No. 8.

### MEDICAL SOCIETY OF THE STATE OF NEW YORK.

THE ninety-second annual meeting of this society was held at Albany, January 25, 26 and 27, 1898, under the presidency of Dr. Seneca D. Powell, of New York. The society on this occasion returned to its old quarters in the city hall, which, on the whole, served quite as well for a meeting place as did the Jermain hall at a cost of $75 a day.

The business committee, consisting of Dr. W. G. Macdonald, chairman, Dr. Ernest Wende, of Buffalo, and Dr. B. Farqhuar Curtis, of New York, had prepared an elaborate program which was disposed of after the usual fashion—namely, hurried reading of papers, limitation of debate, and in some instances failure of authors to appear. An effort had been made to keep the number of papers within a limit that would render the program easily handled during the appointed time. But it appears to be impossible to do this when as now so many readers claim the right to be heard in this great state society.

The president's inaugural address was a model of brevity and conciseness, expressed in forceful and elegant English. He made but few recommendations, some of which were of a negative character. He advised against changing the place of meeting from Albany to other cities in the state ; that no change be made in the method of electing officers ; that better safeguards be thrown around the sale of poisons, dwelling upon the number of cases of carbolic acid poisoning ; reported a list of members deceased during the year ; and commended the work of the state medical examining board.

The principal legislative business of the first morning session involved three questions of importance : first, the redistricting measure proposed by Dr. William Browning, of Brooklyn ; second, a proposal to change the place of meeting from Albany to other prominent cities in the state ; third, and finally, a suggestion to change the method of electing officers, abolishing the nominating committee and taking the subject to the floor. Happily these three propositions were voted down. Every change in the organic law of a society should be justified on grounds of improvement, simplicity, economy or justice. As none of these propositions were sustained by such a foundation, they very properly fell to the ground.

The scientific part of the program dealt with many questions of professional interest. Two papers, at least, were of public concern. Dr. John H. Pryor, of Buffalo, presented one of these under the title, What shall the state and the county do for the consumptive? He pointed out that 13,000 consumptives die annually in the state of New York, and that many might be saved by appropriate care during the incipient stages of the disease. He urged the establishment of a proper home for such persons in the Adirondacks. The essential features of the paper were referred to the committee on hygiene for consideration and report. We understand that resolutions have been introduced in both houses of the legislature inquiring into the feasibility of the plan proposed, on which a hearing has been fixed at an early day. On another page in this issue we make further reference to this important subject.

The other paper of public interest was presented by Dr. J. B. Ransom, of Dannemora, entitled Expert medical testimony. For some time past both the legal and medical professions have been measurably united in the opinion that reform was necessary in this most important field. There has been considerable difficulty, however, in arriving at, or agreeing upon, a proper plan for relief and improvement. Dr. Ransom said that it is now generally agreed that it would be better to have medical experts appointed by the trial judge in each case, and to have some fixed standard of qualification. In another place in the JOURNAL we publish a paper by Tracy C. Becker, Esq., lecturer on criminal law and procedure and medical jurisprudence, in the law department of the University of Buffalo, that may be read with interest and profit as bearing direct relation to this subject. Especially will there be amazement at the difficulties Mr. Becker experienced in obtaining a modification at the hands of the legislature of the present system relating to coroners' juries.

The usual X-ray exhibition appropriated the evening session of the first day. This afforded an opportunity for the enthusiasts on electrical photography to display their ingenuity in reproducing pictorial illustrations of medical and surgical lesions, as well as in demonstrating teaching facilities or possibilities afforded by Röntgen's discovery. We may appropriately remark that this exhibition was unusually attractive and instructive.

The president chose for the subject of his anniversary address, The obligations of the physician and the layman to each other. This was delivered Wednesday evening in the senate chamber as usual. The theme was well handled despite its brevity, and will furnish a subject for much thought. After the address the president entertained the society by a reception at the Albany club, this taking the place of the customary annual dinner. We understand that this was the president's method of entering his protest against a tradition in the society of taxing the president for all deficiencies at the annual dinner. We are glad to know that this question has been raised in this manner and we hope it will lead to an abolishment of the practice.

Happily the society was not inflicted with a discussion on the dispensary bill proposed by the Medical Society of the County of New York. It was evident to the proponents of the measure that it would not receive the endorsement of the society in its present form, hence they very wisely did not raise the issue.

The society nominated, to fill vacancies on the state board of examiners, the following names: Drs. Eugene Beach, of Gloversville, J. P. Creveling, of Auburn, H. D. Wey, of Elmira, and Daniel Lewis, of New York. The state board of medical examiners elected the following officers: president, Dr. William Warren Potter, of Buffalo; secretary, Dr. Maurice J. Lewi, of New York; members of the question committee, Dr. George Ryerson Fowler, of Brooklyn, and the secretary, Dr. Lewi.

The society elected the following officers for the ensuing year: president, Dr. John O. Roe, of Rochester; vice-president, Dr. E. F. Brush, of Mt. Vernon; the secretary, Dr. Curtis, and the treasurer, Dr. Porter, both of Albany, were reëlected.

There are two important improvements, if not reforms, that, it seems to us, this society should institute. The first is that it should appoint its meetings to be held during three full days. There is no good reason that can be offered for the exodus of the members Wednesday evening, leaving comparatively empty benches as an audience for the readers Thursday morning. Other states of far less importance devote three full days to their medical society work. When this society does likewise it will enable itself to dispose of its program in a more satisfactory manner.

The other suggestion we offer is that hereafter the members be assessed for the full cost of each plate at the banquet, or else, if present prices be retained, a deficiency bill be appropriated payable out of the treasury. In any event it is high time for the society to abolish the disgraceful method of taxing the president for deficiencies resulting from its annual feast.

